# Pain from the life cycle perspective: Evaluation and Measurement through psychophysical methods of category estimation and magnitude estimation**^1^**

**DOI:** 10.1590/1518-8345t.0714.2769

**Published:** 2016-08-18

**Authors:** Fátima Aparecida Emm Faleiros Sousa, Talita de Cássia Raminelli da Silva, Hilze Benigno de Oliveira Moura Siqueira, Simone Saltareli, Rodrigo Ramon Falconi Gomez, Priscilla Hortense

**Affiliations:** 2 PhD, Full Professor, Escola de Enfermagem de Ribeirão Preto, Universidade de São Paulo, PAHO/WHO Collaborating Centre for Nursing Research Development, Ribeirão Preto, SP, Brazil; 3 RN, Doctoral Student, Escola de Enfermagem de Ribeirão Preto, Universidade de São Paulo, PAHO/WHO Collaborating Centre for Nursing Research Development, Ribeirão Preto, SP, Brazil.; 4 PhD, Substitute Professor, Instituto Federal de Educação, Ciência and Tecnologia do Sertão Pernambucano, Campus Petrolina, Petrolina, PE, Brazil.; 5 Psychologist, PhD.; 6 Master's Student, Escola de Enfermagem de Ribeirão Preto, Universidade de São Paulo, PAHO/WHO Collaborating Centre for Nursing Research Development, Ribeirão Preto, SP, Brazil.; 7 PhD, Adjunct Professor, Faculdade de Enfermagem, Universidade Federal de São Carlos, São Carlos, SP, Brazil.

**Keywords:** Pain, Pain Measurement, Life Cycle Stages

## Abstract

**Objective:**

to describe acute and chronic pain from the perspective of the life cycle.

**Methods:**

participants: 861 people in pain. The Multidimensional Pain Evaluation Scale (MPES) was used.

**Results:**

in the category estimation method the highest descriptors of chronic pain for children/ adolescents were "Annoying" and for adults "Uncomfortable". The highest descriptors of acute pain for children/adolescents was "Complicated"; and for adults was "Unbearable". In magnitude estimation method, the highest descriptors of chronic pain was "Desperate" and for descriptors of acute pain was "Terrible".

**Conclusions:**

the MPES is a reliable scale it can be applied during different stages of development.

## Introduction

Pain, considered multidimensional^1^ ^)^ and inherent to human existence, is part of the life cycle process. To better understand the process of pain through human development, Piaget's theory is an applicable resource, since it considers the life cycle a continuous construction from a lower to a higher state of balance, preventing the existence of new knowledge without any previous one, in order to absorb and change it, involving intelligent activities, such as assimilation, accommodation and adaptation^2^.

The classical theory of Piaget may support studies about human pain^3^ ^-^ ^4^, since the author studies the human being from birth to adulthood, dividing the cognitive development in sensorimotor, pre-operational, solid operational and formal operational as well linking cognition and affectivity^5^. 

It is important to mention that one of the criticisms against the work developed by Piaget is related to the fact that he considers adolescents (12 to 18 years of age) as equal to adults, meaning that everyone over 12 is considered to have logical formal thinking. Authors^6^ discuss a stage related to adulthood, named Post-Formal Thinking. They describe flexibility, adaptability, openness and individualism as the features of this thinking. These features allow the adults to deal with ambiguous situations, such as contradiction, based on the principle that Complicated situations require Complicated solutions, which means a much more Complicated thinking than the one in the formal operation stage that is characteristic of adolescence.

In a progressive movement, there are the constant functions and the variable structures at six levels or successive stages that involve reflective mechanisms, action, language and thought.

Understanding the language/expression of pain in various situations of the illness/health process becomes a challenge that needs to be overcome and which refers to the way people think, view and express pain caused by the event experienced, especially when they are in different stages of human development.

Piaget's development proposal considers the intellectual function as part of the physiological integrality of the human being. From this theoretical perspective of the life cycle, the concepts of maturation and experience interact, since the presence of certain psychological structures gives meaning to the information captured by the person in each acquisition of experience^7^.

Being pain a phenomenon influenced by cultural and situational factors, and also by care, motivation, emotion and other psychological variables, besides external variables, most information required for a pain evaluation procedure to be appropriate comes from what people think and report, supplemented by physical evaluation^8^.

With proper evaluation, it is possible examining the nature, origins and the clinical correlates of pain, depending on the emotional, motivational and cognitive features, as well as the personality features of patients^8^.

Based on this was developed and validated the Multidimensional Pain Evaluation Scale (MPES), which contains 119 descriptors chronic and acute pain its dimensions: affective/ sensitive and affective/ cognitive. This scale meets the psychometric criteria of reliability, objectivity and consistency^8^. 

The objective of this study was to describe acute and chronic pain from the perspective of the life cycle through the descriptor the MPES. 

## Methods

This is a cross sectional study with a quantitative approach, involved 861 people in pain, between 05 and 93 years of age, 100 being children/adolescents and 761 adult/elderly people. Among these 861 people in pain, 688 presented chronic pain, 647 of whom were evaluated through the category estimation method and 41 through the magnitude estimation method. In addition, 173 people presented acute pain, 125 of them evaluated through the category estimation method and 48 through the magnitude estimation method.

The study was carried out at outpatient care centers and nursing wards at Hospitals and Universities located in country towns of Sao Paulo, Minas Gerais and Paraná.

It was approved by the Research Ethics Committee of the Public Hospital of State Sao Paulo, number 1358/2011, as per Resolution 196/96, related to research involving human beings. 

The MPES was used, encompassing 119 acute pain descriptors and 119 chronic pain descriptors, developed and validated by Faleiros Sousa and contributors^8^.

Prior to the application of the instrument, an Informed Consent Form was provided to the participants to be read and signed. After this procedure, the instrument was applied. A task description was provided at the beginning of the instrument, and the participants were expected to read it before completing it.

The category and the magnitude estimation methods were used. In relation to the category estimation method, each participant's task was to evaluate his/her pain, on a scale from 0 to 10, 0 representing no pain, 10 the highest level of pain, and 1 to 9 intermediate levels of pain. In the magnitude estimation method, each participant's task was to quantitatively evaluate how much a descriptor presents higher or lower scores in relation to another descriptor and, then, the proportions between them were established.

The methods of magnitude estimation and cross-modality matching have its response mode based on line length. Standard stimulus had been previously specified. The task of the participants was to assign a number that was proportional to the intensity of pain felt at every assessment, and also proportional to the standard stimulus applied according to the method used. Thus, if the participant thought that a given pain had twice the intensity of the induced pain, he/she pointed to a number twice as large as the standard stimulus. If he/she judged that a given pain had half the intensity of the induced pain, he/she assigned to it a number that was half the one of the induced pain according to the standard stimulus.

Data were analyzed descriptively and quantitatively, and presented in tables. The arithmetic and geometric averages and the respective standard deviations were calculated.

## Results

According to researchers^6^ and the Brazilian Statute of Children and Adolescents (ECA)^9^ which defines adolescence as the phase between twelve and eighteen years of age, the categories of analysis in this study were divided into children from 5 to 7, 8 to 11 and adolescents from 12 to 18 years of age, while the adults, considered to have post-formal thinking, were allocated into the age group between 19 and 93.

The results related to the use of the category estimation method are described in Table 1, which present the highest and lowest descriptors in the characterization of chronic pain about age group 05-18 years old participants.

**Table 1:**
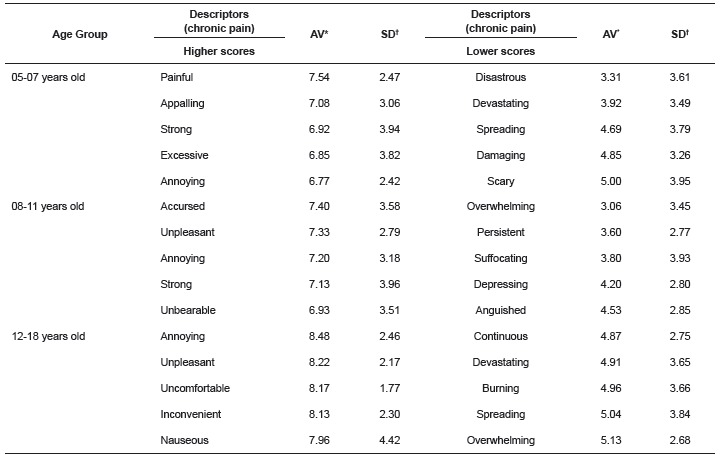
Arithmetic Averages and respective Standard Deviations of characterization of chronic pain in relation to the age group between 05 and 18, according to the MPES. Ribeirão Preto, SP, Brazil, 2012

*AV= Arithmetic Averages, †SD= Standard Deviations.

The results related to the use of the category estimation method are described in Table 2, which also presents the highest and lowest descriptors in the characterization of chronic pain about age group (age 19-93) years old participants.

**Table 2:** Arithmetic Averages and respective Standard Deviations of characterization of chronic pain in relation to the age group between 19 and 93, according to the MPES. Ribeirão Preto, SP, Brazil, 2012

Age Group	Descriptors (chronic pain)	AV^*^	SD^†^	Descriptors (chronic pain)	AV^*^	SD^†^
	Higher scores			Lower scores		
19-93 years old	Uncomfortable	7.74	2.20	Wretched	3.47	3.09
	Unpleasant	7.59	2.24	Demonic	4.69	2.92
	Inconvenient	7.57	2.17	Brutal	3.87	2.52
	Painful	7.49	2.35	Scary	4.07	2.96
	Strong	7.28	2.15	Monstrous	5.12	2.93

*AV= Arithmetic Averages, †SD= Standard Deviations.

The results related to the use of the category estimation method are described in Table 3, which also presents the highest and lowest descriptors in the characterization of acute pain about age group 05-18 years old participants. 

**Table 3:** Arithmetic Averages and respective Standard Deviations of characterization of acute pain in relation to the age group between 05 and 18, according to the MPES. Ribeirão Preto, SP, Brazil, 2012

Age Group	Descriptors (acute pain)	AV^*^	SD^†^	Descriptors (acute pain)	AV^*^	SD^†^
	Higher scores			Lower scores		
05-07 years old	Complicated	8.20	2.34	Fearfull	2.80	2.04
	Moslests	8.00	2.86	Round	2.90	3.24
	Unpleasant	7.80	2.39	Scathing	3.10	3.84
	Crushing	7.70	3.33	Hallucinating	3.80	3.79
	Rampant	7.30	3.12	Debilitating	3.90	2.80
08-11 years old	Unpleasant	6.94	3.47	Overwhelming	1.38	2.65
	Painful	6.81	2.92	Cold	1.69	2.21
	Annoying	6.63	3.09	Desperate	2.13	2.44
	Considerable	6.00	3.55	Shocking	2.25	3.04
	Uncomfortable	5.81	2.88	Hallucinating	2.38	3.09
12-18 years old	Annoying	7.57	2.88	Scathing	2.57	3.40
	Unpleasant	7.13	2.45	Cold	2.70	3.41
	Uncomfortable	5.96	3.25	Desperate	2.74	3.07
	Clear	5.91	3.89	Crazy	3.13	4.05
	Bombarding	5.98	3.25	Destructive	3.17	3.40

*AV= Arithmetic Averages, †SD= Standard Deviations.

The results related to the use of the category estimation method are described in Table 4, which presents the highest and lowest descriptors in the characterization of acute pain about age group 22-69 years old participants.

**Table 4:** Arithmetic Averages and respective Standard Deviations of characterization of acute pain in relation to the age group between 22 and 69, according to the MPES. Ribeirão Preto, SP, Brazil, 2012

Age Group	Descriptors (acute pain)	AV^*^	SD^†^	Descriptors (acute pain)	AV^*^	SD^†^
	Higher scores			Lower scores		
22 - 69 years old	Unbearable	6.73	2.60	Maddening	2.03	1.07
	Intense	6.32	2.71	Blinding	2.94	1.29
	Desperate	5.87	2.37	Hallucinating	3.09	1.91
	Terrible	5.64	2.81	Annililate	3.58	2.10
	Tremendous	5.23	2.50	Inhumane	3.9	2.07

*AV= Arithmetic Averages, †SD= Standard Deviations.

Concerning the analysis of the 100 children/adolescents who participated in the study, 51% recorded chronic pain and 49% acute pain. The data in Table 1 show the characterization of chronic pain and in Table 3 the characterization of acute pain according to the MPES, for the age groups related to childhood and adolescence, which are subdivided into three stages of development.

Related to adult population, in Table 2 the data show the descriptors with highest and lowest scores according to the MPES for the characterization of chronic pain and in the Table 4, the characterization of acute pain. The arithmetic averages (AV) and respective standard deviations (SD) are listed in the table. 

Table 5 presents twenty acute pain and twenty chronic pain descriptors, in order of position and with the respective Geometric Averages (GA), which were obtained through the magnitude estimation method.

**Table 5:** Geometric Averages of characterization of acute and chronic pain through the psychophysical Magnitude Estimation Method. Ribeirão Preto, SP, Brazil, 2012

Position Order	Age group	Descriptors of chronic pain (n=41)	GA^*^	Age group	Descriptors of acute pain (n=48)	GA^*^
1	21 to 92 years old	Agonizing	446.31	14 to 70 years old	Terrible	115.56
2		Cruel	389.89		Strong	113.78
3		Harmful	352.27		Unbearable	111.92
4		Terrible	330.04		Intense	110.35
5		Discomforting	279.11		Violent	104.22
6		Disastrous	276.17		Deep	103.70
7		Intense	262.29		Monstrous	100
8		Unbearable	254.93		Dreadful	98.17
9		Depressing	254.93		Desperate	88.66
10		Concerning	254.08		Maddening	77.71
11		Painful	249.61		Tremendous	75.90
12		Aggressive	237.91		Brutal	75.61
13		Tiring	228.95		Inhumane	71.72
14		Anguished	208.96		Annililate	70.72
15		Suffocating	208.13		Lacerating	68.36
16		Uncomfortable	204.33		Blinding	68.22
17		Nauseous	201.04		Hallucinating	67.30
18		Strong	198.80		Fulminat	64.60
19		Disturbing	192.86		Overwhelming	62.46
20		Annoying	188.22		Colossal	59.32

*GA = Geometric Averages.

## Discussion

Considering the multidimensional features of pain that the descriptors of the MPES carry, predominantly in conjunction with affective components and sensitive components, while others contain the cognitive component, this dyads affective/ sensitive and affective/ cognitive occur because the pain is seen from the view point of emotions^1^ ^-^ ^8^.

The phenomenon of existence is immersed in the emotions and affections that the person experiences, thus its expression as pain is crossed by the emotional dimension, being conceived and lived through this perspective^10^ .

When the subject reports a pain descriptor, he/she not only reports the contents of pain, but also all emotional content present in his relationship with pain and with all the pain contained in relations between people involved in the disease. In other words, in human communication all content transmitted is related to the relationship between individuals^11^.

The results obtained in relation to the contingency of the children's answers show that, in different age groups, the details and logic elements expressed in relation to the descriptors of chronic and acute pain reveal that from the age of five, children can already give meaning to the painful event in a categorical way. In this study, for example, children younger than 7 showed evidences of accurate meanings and verbalizations of pain, suggesting cognitive maturity, thus showing the possibility of development of thinking about pain as a result of their experiences, from the perspective of the development in the life cycle.

Children aged between 5 and 7 characterized the chronic pain dimensions mostly related to the affective/sensitive than to the affective/cognitive dimension. It is understood that this type of response to pain may be related to the fact that children have concrete thinking^12^.

It can be considered that, in order to recognize their pain, children tend to develop a type of thought focused on feelings of their own body, consistent with the literature affirming that the thoughts of children are absolute, and it is difficult to change the belief about the pain as a physical dimension^13^. 

A qualitative study pointed out that children in the pre-operating stage of development described their pain emphasizing sensitive and evaluative aspects, which may indicate the characteristic of this phase in which the children have premature symbolic representation^3^.

In the age group between 8 and 11, there was predominance of the affective/cognitive pain dimension. At this stage, the egocentric thought of the children is developed towards logical thinking and reversibility. The linguistic entities are more extended and operating^12^. 

A transition in terms of development of the children when distinguishing internal from external was verified, extrapolating from the perception of a more sensory pain to another, in which they are able to use qualitative terms, such as the affective and cognitive dimensions. In this situation the dynamic relationships of the family are fundamental for the development of the children and their response to pain^14^.

The perception of pain by adolescents aged between 12 and 18, the affective/cognitive dimension presented higher scores, and lower scores were attributed to affective/sensitive descriptors. The capacity of introspection and the understanding about pain shown by the adolescents should be highlighted, insofar as they are able to disregard the affective/cognitive pain associated with psychological suffering^12^ ^-^ ^13^.

In the analysis by age group, the results regarding the chronic characteristics showed heterogeneity in the responses attributed to pain. Thus, the children aged between 5 and 7 stressed the affective/sensitive dimension, significantly representing the concrete thinking, those aged from 8 to 11 stated the quality of the pain in the affective/cognitive dimension, representing the transition from the concrete to the abstract thinking; and the adolescents viewed the pain mediated by its affective/cognitive nature, representing the complementarity of the logic-formal abstraction described by Piaget^4^ ^,^ ^12^.

Studying the pain of children/adolescents studies show that from the age of 4, children are capable of describing their pain through sensitive descriptors in the same way as it is described by older children or adults^3^ ^,^ ^15^.

In the age group between 19 and 93, the descriptors that best characterized chronic pain were descriptors in the affective/sensitive dimension, whose expression refers to the physical sensation of the pain event, also characterizing the affective/cognitive dimension of pain, in which there is, in addition to the emotional nature, explanation, rationalization and intellectualization of the perceived event. 

A study aimed to evaluate the sensitive, affective, temporal and miscellaneous qualities of pain in elderly people with chronic pain, provide information about the different perceptions of the various factors that are part of the pain symptom, in relation to people with different diseases, since the perception of this symptom is related to sensitive, affective and motivational aspects, and not only to the intensity^16^. Another study shows predominance of chronic pain in elderly people, highlighting the prejudices to the quality of life due to physical and affective dimension of pain, such as inactivity and social isolation^17^. 

When compared the characterizations of acute and chronic pain, in all age groups of children/adolescent, the descriptors used to characterize acute pain were mainly related to the affective/cognitive dimension, with little change among the age groups; in contrast, the descriptors used to characterize chronic pain showed procedural thinking, i.e., showing changes throughout the development.

The results did not only show differences, but they also revealed an important similar detail in relation to the descriptors 'annoying' and 'unpleasant', which were consistent in the attributions for both acute pain and chronic pain. Such information may reinforce the presence of affective and cognitive components in the pain responses by the children and adolescents in this study, representing the continuous, complicated development of abstract thinking.

A study brings up the discussion about affection in the organization of human thinking and understanding about the interconnection between the cognitive and affective dimensions, showing that human beings not only build logic and rational relations between the external and the internal reality, nor are they only emotional in relation to feelings and states of mind, but a dynamic sum of these two dimensions, as cognitive-affective being who both think and have feelings and affections^18^.

A study with children and adolescents showed to be interconnected with the application of the MPES in the present study, showing the use of pain evaluation instruments with the possibility to ensure the understanding about what children really experience, and not what the professionals think they are feeling. Based on this, it is necessary to consider the processes concerning the experience of the children, as well as their stages of physical and mental development^19^.

As for the age group between 19 and 93, the descriptors that best characterized acute pain were affective/cognitive, similar to a study developed to evaluate pain in cancer patients, that showed that the most frequently mentioned descriptors were those with the sensitive component, but the ones with higher scores were those with the affective component^20^. Therefore, the age group between 19 and 93 highlighted the affective and cognitive dimensions, although the sensitive dimension was present, showing a more complicated thinking that takes into account, but goes beyond the physical aspects, considering other aspects involved in the understanding of the pain event.

In relation to the magnitude estimation for chronic and acute pain, the five descriptors with highest and lowest scores in the characterization were mostly in the affective/cognitive dimension. 

Studies that evaluated both chronic pain and acute pain using the psychophysical method of magnitude estimation, had results indicating that the affective/cognitive dimension is the most used in the characterization of pain^21^ ^-^ ^22^. 

When comparing the mentioned data with those from this study, the importance of the affective and cognitive dimensions can be noted for people experiencing pain. Therefore, health professionals evaluating and dealing with patients during their painful experience, cannot neglect these dimensions. Through these data, it can be identified that the perception of adult human beings considers a range of elements that constitute their pain. Besides its physical aspect, this experience is loaded with affection and, both in chronic and acute pain, thoughts are focused on understanding it, in a way that occupies patients' minds needing development at the level of ideas and affections.

The participants, who scored the affective dimension of pain highest, agree with the expectations of dealing with their own pain, immersed in the universe of symbolic meaning and attribution of subjective qualities. Such subjective qualities are intertwined with people, objects and places of experiences. Thus, the psychological subject "returns" the result of his mental action in evaluative quality^23^.

It shows that it is not possible to understand the pain experience objectively or to perceive it as a universal conceptual entity, since it is a personal experience, and it is through the language that the specific characteristics of each painful sensation can be expressed, and they significantly differ one from another^8^ ^,^ ^24^. 

Either in the acute or chronic characterizations, pain is viewed as "total pain", because it in addition to the nociception, the physical, emotional, social and spiritual factors, among others, affect the genesis and painful expression. Pain evaluation is Complicated, due to the variety of aspects that compose the pain event, and it is the basis for the diagnosis, the treatment proposal and the assessment of the obtained results^25^.

## Conclusion

In summary, when analyzing the results of this study, it is observed that people need to be viewed in an integrative manner, while developing people, with a history, cognition, desire and affection cannot be ignored. All these attributes are resources that need to be considered for a more complete evaluation going beyond pain intensity, in other words, a multidimensional evaluation of the total pain experience, being worth of note that the MPES is validated and an easy instrument to apply in different stages of development.

This study can contribute to show an emerging reality of evaluation of the fifth vital sign that cannot be underestimated, since the healthcare team in general needs to realize the complexity and multidimensionality existing in the pain of existing in the world in different situations and subjective perspectives, and this is a reality that needs to be reconsidered with a view to possible improvements in education, research and clinical practice.
